# Feelings of guilt and pride: Consumer intention to buy LED lights

**DOI:** 10.1371/journal.pone.0234602

**Published:** 2020-06-25

**Authors:** Sedigheh Moghavvemi, Noor Ismawati Jaafar, Ainin Sulaiman, Farzana Parveen Tajudeen

**Affiliations:** Faculty of Business and Accountancy, University of Malaya, Kuala Lumpur, Malaysia; University of Maryland College Park, UNITED STATES

## Abstract

The adoption of energy-efficient lighting systems such as light-emitting diode (LED) lights is an effective strategy to address global warming and climate change. The adoption of LED light usage is largely shaped by consumer behavior. Understanding the factors that influence consumers’ awareness of the advantages of using LED lights and consumers’ eventual buying behavior is critical to industry players and policy-makers. The objective of this study is to investigate the significant factors that affect consumer intention to use LED lights among Malaysian households. A survey questionnaire of 1,075 potential consumers was employed and the partial least squares technique was applied in data analysis. The results show that consumer awareness creates responsibility and influences consumers’ personal norms and attitudes, and their ultimate intention to buy LED lights. Feelings of guilt about contributing to air pollution and an awareness of the advantages of using LED lights both activate individual personal norms and attitudes, and influence the intention to buy LED lights. Awareness of the advantages of using LED lights, and of the negative consequences of not using them, prompts consumers to have feelings of guilt. The findings point to the importance of creating awareness among consumers.

## Introduction

Energy conservation and environmental preservation are global issues receiving widespread attention. Undesirable human behavior has propagated global environmental challenges including biodiversity loss, resource depletion and climate change [1] that endanger humankind. The consumption of fossil fuels has increased the concentration of greenhouse gas emissions, causing the atmosphere and oceans to become warmer, melting high amounts of snow and ice, and raising sea levels [2, 3]. Sustainable development is not possible without sustainable sources of energy and responsible behavior of individuals towards the environment.

Human behavior broadly, and the feeling of a responsibility to perform specific behaviors in particular, are among the determinants of sustainable development which influence the environment. The feeling of moral obligation and other emotions can potentially influence environmental behavior. In the context of human behavior, individuals are capable of anticipating the emotions related to future outcomes [4]. The association with human behavior points to the complexity of sustainable development and the need for comprehensive planning. For example, anticipated emotions are found to influence individuals’ behavior and affect their decision-making [5]. Therefore, understanding the effect of anticipated emotions is an important factor in understanding individual decision-making such as the intention to buy light-emitting diode (LED) lights.

Previous studies have mainly examined the impact of anticipated emotions on individual decision-making based on the theory of planned behavior [6]. The studies showed that anticipated emotions do not affect behavior directly but through variables such as intentions and behavioral expectations [4], and that anticipated emotions such as pride and guilt are related to individual decision-making about the environment [7].

The emotions of pride and guilt arise when people compare their behavior with a set of social or personal norms that determine the wrong or right action [4]. These emotions (such as shame, hubris, embarrassment) are evoked by evaluations of one’s self after following or failing to follow personal or social standards [8]. The violation of personal norms evokes feelings of guilt, while compliance stimulates feelings of pride. Anticipated guilt is formed in interpersonal contexts and arises from a perceived mismatch between one’s own behavior and social norms; while anticipated pride increases self-control and affects behavior over time [9]. Research shows that anticipated guilt and anticipated pride, which are formed on the basis of an individual’s moral obligations, have a significant influence on pro-environmental intentions [4]. Pride and guilt are self-conscious emotions which play a distinct role in an individual’s decision-making process and are relevant in understanding pro-environmental behavior.

According to the norm activation model (NAM), people are more likely to reduce their energy consumption when they feel morally obligated to do so [10]. Norm activation begins with an individual’s awareness of consequences and a sense of responsibility for the consequences of not acting pro-environmentally. This awareness will determine whether the individual feels they should perform a particular action that prevents a harmful outcome [11]. If they believe that energy saving and pro-environmental behavior is moral and valued then they are likely to feel proud of this behavior, whereas a lack of engagement when opportunities arise should result in feelings of guilt [12]. Pride in environmental behavior will influence individual engagement in pro-environmental behavior [12]. Harth et al. [7], for example, found that anticipated pride about in-group pro-environmental behavior stimulated the desire to donate money for environmental protection.

Current energy consumption patterns and related environmental pressures have ignited concerns about energy conservation among many governments, including the Malaysian government. Malaysia’s electricity system operator, Tenagah National Berhad (TNB), promotes the transition towards a low-carbon economy and improve energy efficiency. The energy ministry has committed to retrofit 50 government buildings with energy-efficient LED lightings. Six of TNB’s distribution buildings were selected as models of energy-efficient buildings in the country. The government, housing development and town and country planning agencies announced that Penang is targeted to be the first state in Malaysia to use energy-efficient lamps for all street lighting by 2020 as part of the green agenda to reduce Malaysia’s carbon footprint [13].

There is a strong global movement towards the use of more efficient lighting. It is predicted that LED and compact fluorescent lamps will replace 90% of the world’s indoor lighting by 2022 [14]. However, the usage and acceptance of the LED light depends on the individual, their awareness, decision-making and lifestyle, and related environmental issues. Therefore, understanding the individual’s approach to decision-making is crucial to understanding usage behaviors and the connection to environmental issues [4]. A growing number of consumers engage actively in green shopping practices as a way of fulfilling their sense of commitment towards the environment [15]. To date, however, researchers have paid little attention to the discovery of the significant factors affecting the individual’s adoption and usage of energy-efficient lighting technologies such as the feeling of anticipated guilt and anticipated pride among individual consumers.

The aim of the present study is to identify the factors affecting the intention of the consumer to buy LED lights. At the foundation of this research is the understanding of pro-environmental behaviors (including LED usage) as a component of pro-social behaviors (often associated with morality) [16, 17] whereby individuals are influenced to act pro-socially to benefit others or themselves [18]. This study employed the NAM [11] as its theoretical foundation. As well as identifying the factors that affect the intention of the consumer to buy LED lights using the NAM, the study also investigates the influence of anticipated emotion on consumers’ usage intentions towards LED lights. Researchers found three decades ago that emotions have an important impact on environmental behaviors [19], yet it is only recently that empirical studies have investigated the role of emotions in the decisions made by individuals to act in more environmentally-friendly ways [12]. Consequently, the present study set out to examine the role of ‘anticipated pride’ and ‘anticipated guilt’ in positive environmental behavior guidance within the framework of the NAM. The study extends the classic version of the NAM by adding ‘attitude’ as an intermediary variable, ‘intention to use’ as a dependent variable, and ‘anticipated guilt’ and ‘anticipated pride’ as moderator variables.

## Literature review

Global warming and climate change issues have caused policy-makers to pay urgent attention to global sustainability programs. Policy-makers around the world have developed programs to adopt energy efficiency technologies and practices to decrease energy consumption. These programs started as early as 1970 with many countries devising energy efficiency policies to address the 1970s’ oil price shocks, resulting in a decline in total energy demand by 1998. This result is commendable as the total energy demand in 1998 would have been 50% higher without these policies and ensuing practices [20]. Households in many major economies avoided additional spending of close to US$300 billion on energy in 2016 as a result of energy efficiency policies in place since 2000. Energy-saving equipment, appliances and lighting products facilitate energy savings of around 10% to 20% in most countries [14].

About 20% of the total power usage in the world is from lighting [21]. In some countries such as Malaysia, the air conditioning and lighting consumption is the highest component of a building’s total energy consumption due to the country’s hot climate conditions. Malaysia’s energy consumption levels for air conditioning, refrigeration and lighting are 8%, 26% and 25% of the country’s total energy consumption, respectively [22]. Compounding this high consumption is the fact that less than 11% of Malaysia’s electricity is produced using non-renewable energy resources, with the bulk of the country’s electricity production dependent on coal and natural gas (89% in 2015) [23]. Although the share of natural gas will remain unchanged by 2030, it is alarming that the share of coal is predicted to increase to 49% [24].

High usage of fossil fuel resources is the main cause of climate change and global warming. Considering the continual high use of energy and the depletion of fossil fuel sources, energy conservation policies remain one of the best ways to ensure the supply of adequate energy to meet future demand and to alleviate climate change. Consequently, energy-saving policies have become vital in recent years [25]. The International Energy Agency stated that efficiency improvements in energy consumption can reduce 31% of emissions and that it is necessary to halve the 2009 levels of energy emission by 2050 [26].

Although lighting usage behavior is dependent on culture and differs across nations and regions, the benefits of energy conservation and environmental preservation policies are accepted globally [27]. Despite its low luminous efficiency, short life and adverse impact on the environment, incandescent lighting is still widely used around the world as a source of lighting for buildings [28]. Electricity consumption can be reduced by energy-efficient lighting technologies such as LED lighting.

LED lights were initially used in numeric and alpha-numeric displays and gradually were applied in the automotive industry and in devices such as traffic lights, backlights and flashlights for general lighting and mobile devices [29]. LED lighting is now among the rapidly growing innovations in energy-efficient technologies for lighting which can contribute to decreases in energy usage, alleviate climate change and promote the viable functioning of cities in the long term [30]. The adoption of LED technology is expected to reduce the levels of energy used for lighting because the LED bulb absorbs 50% less power than fluorescent lighting [25]. LED lights are energy-efficient illumination lights with longer lifetimes and better design flexibility. In comparison to fluorescent lights, LED lights have 9 to 10 times longer lives [31]. LED lights are capable of generating more light and less heat compared to fluorescent and incandescent lights. In addition, unlike fluorescent lights, the size and shape do not influence the performance of LED lights [32]. In view of the energy efficiency advantages of LED lights, it is useful to investigate the effective ways of promoting consumer adoption of LED lights and to analyze the factors that influence the attitudes of consumers and their desire to purchase LED lights.

## Hypotheses development

Socio-psychological theories have widened the understanding of pro-social behaviors [33]. In the literature, the NAM [11] has been used to investigate different types of pro-social desires and behaviors, such as the donations of bone marrow or blood [33]. The model is used in the context of pro-environmental behaviors to investigate anticipated pride and guilt. The NAM is commonly used for studies on pro-social behaviors and altruistic predictions [34]. Pro-social behaviors encompass a wide variety of activities that assist others including cooperating and sharing [35, 36]. Pro-environmental behavior has beneficial effects for others and is often regarded as a type of pro-social behavior [33].

The core of the NAM is personal norms. According to the NAM, once an individual has a moral obligation, they will engage in an altruistic act as their personal norm has been activated [11]. The efficiency of applying the NAM in the context of pro-environmental behavior studies has been demonstrated by researchers such as Klöckner [1] and Onwezen et al. [4]. The NAM provides three elements to forecast pro-social behavior, namely, awareness of impact, responsibility obligation, and personal norms. The awareness of impact relates to knowledge of the negative implications of not acting pro-socially. The responsibility obligation refers to a sense of duty to avoid the adverse effects of not acting pro-socially [37]. Awareness of impact and the consideration of accountability predict the personal norm of an individual which is the feeling of “moral obligation to perform or refrain from specific actions” [38].

**Fig 1:**
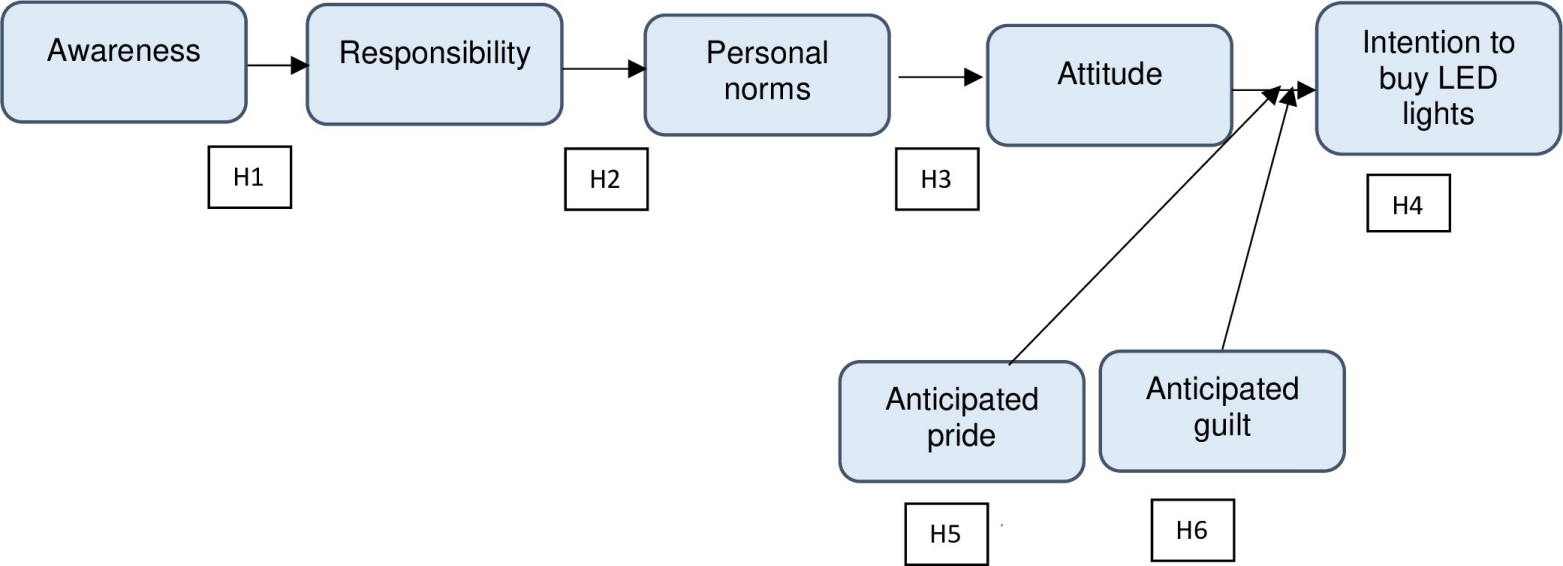
Theoretical framework: NAM and anticipated emotions.

The personal norm is the immediate determinant of pro-social behavior that is activated by an individual’s awareness of the impact of performing or not performing a certain act as well as the feeling of responsibility for the impact [11]. In the process of a norm activation, the personal norm is the main element in enhancing an individual’s implementation of environmentally appropriate behavior and decision-making. When a personal norm is activated, it affects intention and behavior [36, 39]. This research assumes that the realization of the impact will inspire accountability that in turn activates the individual norm (Fig 1). The following hypotheses were developed on the basis of these assumptions:

H1: The awareness of consequences has a significant influence on the acceptance of responsibility to use LED lights.
H2: The acceptance of responsibility has a significant influence on consumers’ personal norms.

### Attitudes

The NAM concentrates on individual norms and overlooks the function of variables such as attitudes, intentions, the situation itself and habits [40]. The extant literature highlights that the integration of volition and subjective attitude in the NAM illuminates the understandings of an individual’s eco-friendly behavior [1, 41]. The attitude of an individual towards a certain conduct relates to the negative or positive tendency to consistently react to a particular activity [42].

Behavioral beliefs that are invoked throughout a specific scenario shape attitude. Attitude is an individual’s assessment of the favorability of a behavioral alternative [1]. Eagly and Chaiken defined attitude as an individual’s assessment about whether or not to conduct instrumental behavior, and they found that attitude indicates a person’s predisposition to react with particular behaviors [43]. Attitude as the cause of specific behavior is frequently evoked automatically as the individual is subjected to particular action [44]. Attitude determines conduct indirectly through behavioral intention [41].

The theory of planned behavior proposes that intention is the closest determinant of behavior and is itself predicted by attitude toward perceived behavioral control, subjective norms and behavior. As intention may not always lead to behavior, it is a slightly stronger dependent measure than a behavioral measure. However, many studies have found that intention predicts behavior most of the time [37, 45]. Studies have revealed that, when the NAM is extended to include intention, the explained variance in behavior is significantly increased (by almost 17%) [41, 46]. In addition, it is proposed that a feeling of responsibility activates attitudes and social norms as much as it activates personal norms, a component of NAM [11].

Following the guidance of the extant literature, this study extends the NAM by including attitude and intention to purchase LED lights as variables. Since attitude is a direct determinant of intention, and a feeling of responsibility activates attitude, the following hypotheses were developed in this research to explore the mediating function of attitude towards the association between responsibility and the intention to buy LED lights (Fig 1):

H3: Consumer’s personal norms have a significant influence on positive attitude towards using LED lights.
H4: Positive attitude has a significant influence on the intention to buy LED lights.

### Anticipated emotions

Emotions are evoked as individuals assess their conduct with regard to a set of private or social norms [47, 8]. Lewis [47] categorized self-conscious emotions as the conditions evoking these feelings. Two self-conscious emotions, namely, guilt and pride, become the specific focus of the study as these feelings seem particularly relevant in terms of environmentally-friendly behavior [9].

The ability of feelings of pride and guilt to support modal behavior has been reported repeatedly in social psychology [48, 49]. It is suggested in the literature that negative moral feelings can encourage environmentally-friendly behavior [4, 50]. Guilt is an adverse emotion that makes the person feel liable for an adverse result [48, 51]. The emotion that promotes ethical conduct is called pride because it is a favorable sensation that improves the motivation of customers to act in accordance with personal norms [49, 52].

Studies have shown that anticipated pride and guilt encourage people to act according to environmental social standards and attitudes, hence highlighting the self-regulatory characteristics of these feelings [49]. Based on these assumptions, this research was designed to explore the mediating effect of anticipated pride and guilt on the relationship between attitude and intention to buy LED lights. Previous studies including research by Onwezen et al. [53] and Su et al. [54] investigated the mediating impact of anticipated emotions on the relationship between attitude and intention and only a few research studies have tested the moderating role of these two anticipated emotions on how anticipated feelings improve the intentional effect of attitudes. Accordingly, the following hypotheses were formulated:

H5: The relationship between attitude and intention to buy LED lights is moderated by anticipated pride.
H6: The relationship between attitude and intention to buy LED lights is moderated by anticipated guilt.

## Methods

### Measurement

The measurement items used in this research were adopted from prominent behavioral studies. Items on anticipated guilt were adopted from Jones and Kugler [55]; the guilt inventory, awareness, responsibility and personal norm items were adopted from Schwartz [11]; attitude items were adopted from Ajzen and Fishbein [39]; and anticipated emotion items were adopted from Tracy and Robins [49] (Table 1). To evaluate the items, the research developed a five-point Likert scale. The survey questionnaire comprised two parts. The first part covered respondents’ demographics, and the second part covered the measurement items of the proposed model. In addition, an explanation and photos of LED lights were provided on the cover of the questionnaire to enhance respondents’ understanding.

**Table 1:** Measurement items adopted from the literature.

Researchers	Adopted Measurement Items
Schwartz (1977)	Awareness, Responsibility and Personal Norm
Kugler And Jones (1993)	Inventory of Guilt
Ajzen And Fishbein (1980)	Attitude
Tracy And Robins (2007)	Pride Scales

### Sampling procedure

University Malaya ethical committee approved this research. A questionnaire survey was conducted among 1,300 Malaysian households in states in five regions, namely, Central, North, South, East Malaysia and the East Coast of Malaysia. Records of LED light household users were not available. The questionnaires were distributed to households in major cities because it was assumed that most urban households would have disposable income and could afford to buy LED lights. The sampling frame only represented the samples who might have been willing to buy LED lights. Listings of high-density residential populations were obtained from the local municipal councils of five major cities. Field workers were employed to collect data for the study. Respondents were recruited using the door-to-door method, where only one respondent was selected to complete the questionnaire for each house.

Of the 1,300 respondents surveyed, 1,215 responded (a response rate of 93%) with 1,075 questionnaires accepted as valid. Analysis of the demographics of the survey data (Table 2) reveals that 54% respondents were female and 46% were male. The majority of the respondents (almost 59%) were in the below 30 age group, 22% were in the 31–40 age group and the remaining 19% were in in the ‘other’ age group (above 41). In regard to education, 58% of the respondents were educated up to diploma level with the remaining respondents possessing tertiary education. In regard to the total household monthly income, most of the respondents (57%) reported an income of more than RM3000 (US$717), and 43% of the respondents reported an income of RM3000 (US$717) or less. In terms of household size, 76% of the respondents lived with four or more persons in different types of accommodation comprising a single story dwelling (40%), double story dwelling (25%), and other types of accommodation (35%).

**Table 2:** Demographics of study participants.

Demographics	Classification	Percentage (%)
**Gender**	Female	54
	Male	46
**Age Group**	Below 30	59
	31–40	22
	41 and above	19
**Education Level**	Diploma and below	58
	Tertiary	42
**Income Level**	More than RM3,000 (US$717)	57
	RM3,000 (US$717) or less	43
**Household Size**	4 or more persons	76
	Less than 4 persons	23
**Type of Accommodation**	Single story dwelling	40
	Double story dwelling	25
	Others	35

## Data analysis and results

According to Chin et al. [56], an analysis of interactions and moderations is more effectively conducted with the use of the partial least squares (PLS) technique than with other methods such as covariance-based structural equation modeling. The present study used the PLS technique to evaluate the six hypotheses including the moderating effects of anticipated emotions and the validation of the measurements. The estimated interactions for both the latent variables and their products (outer loadings) were evaluated in order to create the assessment model. Significant outer loadings of higher than 0.708 allowed the retainment of an item in the measurement model, and outer loadings of 0.40 to 0.70 allowed for the removal of an indicator, with the latter applied only if the composite reliability and average variance extracted (AVE) increased [57].

### Assessment of measurement model

The findings revealed that most of the items’ outer loadings exceeded 0.708. Two items had low loadings and were removed from the model, namely, Item AW2 “The effects of pollution from conventional lights (normal lights) on public health are worse than we realize” (0.565) and Item BI6 “If I find that LED lights/lamps are more expensive and difficult to get, it would stop me from buying and using them” (0.222). This improved the composite reliability and AVE. For further assessment, all remaining items were preserved in the model. Table 3 presents the outer loadings of each indicator.

**Table 3:** Factor loadings.

Indicator	Outer loading	Indicator	Outer loading
*AT1 (Attitude)*	0.800	P1 (Anticipated Pride)	0.863
*AT2*	0.732	P2	0.887
*AT3*	0.831	P3	0.887
*AT4*	0.808	P4	0.813
*AW2 (Awareness)*	0.689	P5	0.823
*AW3*	0.718	PN1 (Personal Norm)	0.686
*AW4*	0.753	PN2	0.786
*AW5*	0.730	PN3	0.771
*AW6*	0.702	PN4	0.743
*AW7*	0.688	R1 (Responsibility)	0.768
*BI1 (Behavioral Intention)*	0.839	R2	0.718
*BI2*	0.863	R3	0.638
*BI3*	0.857	R4	0.776
*BI4*	0.770		
*BI5*	0.715		
*G1 (Anticipated Guilt)*	0.887		
*G2*	0.918		
*G3*	0.875		
*G4*	0.878		
*G5*	0.866		

Next, to check for convergent validity and construct reliability, the composite reliability and AVE tests were carried out. The results showed that the values of the composite reliability for all constructs were higher than 0.7 and the values of the AVE were greater than 0.5.Therefore, there was no issue with convergent validity and construct reliability. The results of the composite reliability and AVE are presented in Table 4.

**Table 4:** Construct reliability and convergent validity.

	Composite Reliability	Average Variance Extracted
Anticipated Guilt	0.948	0.783
Anticipated Pride	0.931	0.731
Attitude	0.872	0.630
Awareness	0.861	0.509
Intention	0.905	0.657
Personal Norm	0.835	0.559
Responsibility	0.817	0.528

The discriminant validity was confirmed. Examination of the cross loadings (S1 Appendix) revealed that the factor loadings were greater than other constructs. The construct for the AVE square root was higher than many other correlated values which proved the accomplishment of the discriminant validity. Table 5 presents the results of the Fornell–Larcker test.

**Table 5:** Fornell–Larcker criterion.

	Anticipated Guilt	Anticipated Pride	Attitude	Awareness	Intention	Personal Norm	Responsibility
**Anticipated Guilt**	0.885						
**Anticipated Pride**	0.539	0.855					
**Attitude**	0.231	0.347	0.793				
**Awareness**	0.222	0.237	0.384	0.713			
**Intention**	0.309	0.378	0.519	0.259	0.811		
**Personal Norm**	0.365	0.390	0.539	0.468	0.541	0.747	
**Responsibility**	0.313	0.353	0.482	0.473	0.430	0.586	0.727

The value in the diagonal is the square root of the variance

Henseler, Ringle and Sarstedt stated that the Heterotrait–Monotrait (HTMT) ratio developed in simulation studies is superior in detecting the lack of discriminant validity [58]. Discriminant validity is established for a reflective construct if the value of the HTMT ratio is below 0.90, as indicated by Henseler et al. [58]. The results of the present study showed that the HTMT ratio for all the constructs was below 0.90, thus confirming the discriminant validity. The HTMT values of the constructs are presented in Table 6.

**Table 6:** Heterotrait–Monotrait ratio.

	Anticipated Guilt	Anticipated Pride	Attitude	Awareness	Intention	Personal Norm	Responsibility
**Anticipated Guilt**							
**Anticipated Pride**	0.579						
**Attitude**	0.263	0.405					
**Awareness**	0.254	0.273	0.470				
**Intention**	0.343	0.424	0.618	0.298			
**Personal Norm**	0.437	0.472	0.693	0.599	0.673		
**Responsibility**	0.377	0.426	0.619	0.600	0.533	0.796	

### Structural model evaluation

The structural model evaluation showed that Hypotheses 1 to 4 were supported with a t-value >2.58. It was found that awareness of the consequences of using LED lights created a sense of responsibility to use LED lights (H1). Responsibility, in turn, affected the personal norms towards the use of LED lights (H2). Personal norm subsequently affected the attitude towards LED lights (H3) and attitude influenced the intention to buy LED lights (H4). Table 7 presents the results of the path coefficients and t-values for Hypotheses 1 to 4. The results suggest that creating awareness will influence consumer attitude and intention to buy LED lights.

**Table 7:** Hypotheses.

Hypothesis	Beta	t-value	Result
**H1. There is a significant relationship between awareness and responsibility towards LED lights**	0.618	17.989***	Supported
**H2. There is a significant relationship between responsibility and personal norm towards LED lights**	0.800	28.310***	Supported
**H3. There is a significant relationship between personal norm and attitude towards LED lights**	0.699	22.066***	Supported
**H4. There is a significant relationship between attitude and intention to purchase LED lights**	0.531	14.959***	Supported

*** p<0.01 (>2.58)

**p<0.05 (>1.96), p<0.10 (>1.64)

### Moderation analysis

The results of the moderation analysis (Table 8) show that anticipated pride may not reduce the relationship between attitude and intention to purchase LED lights, with a t-value of <1.96. Therefore, Hypothesis 5 was not supported. The relationship between attitude and intention to purchase LED lights was moderated by anticipated guilt, with a t-value of >1.96. Therefore, Hypothesis 6 was supported. The results suggest that consumers may have a feeling of guilt if they do not buy LED lights as they are aware of the consequences of not using this type of lighting. However, buying LED lights does not give them a feeling of pride.

**Table 8:** Moderation analysis.

Hypothesis	Moderation (t-value)	Result
H5: The relationship between attitude and intention to purchase LED lights is moderated by anticipated pride.	1.119	Not supported
H6: The relationship between attitude and intention to purchase LED lights is moderated by anticipated guilt.	2.079	Supported

*** p<0.01 (>2.58), **p<0.05 (>1.96), p<0.10 (>1.64)

## Discussion

The primary objective of the research was to identify the factors affecting the intention of consumers to purchase LED lights. For the purposes of the study, the NAM was adapted to include the anticipated emotions of guilt and pride as variables. Analysis of the survey results demonstrated the positive impacts of awareness on responsibility towards the usage of LED lights which in turn influenced the personal norm towards the use of LED lights. The findings support the conclusion reached in previous studies in the literature that awareness of impact has an influence on ascribed responsibility and on individual norms [4]. For example, the results of the present study are in line with the findings by Han and Hyun [59] and Zhang et al. [60] that a person will feel accountable for behaving in an environmentally-friendly way when they are aware of the negative impacts of their actions if they do not behave pro-environmentally. This shows the importance of creating awareness among public about the importance of using energy efficient product. Shift public opinion from other energy to renewable energy will remain the key to drive LED industry growth.

The results also showed a significant positive relationship between individual norms and attitudes towards the use of LED lights. The outcome of the study is consistent with the finding by Chen and Chai [61] that there is an important relationship between consumers’ individual norms on environmental issues and their attitudes towards green products. The study found that personal norms affected attitudes towards buying LED lights. Personal norms relate to the consumer's sense of moral obligation, making it a strong motivator for environmental behavior [19, 61]. If a consumer feels morally bound to preserve the environment, it will subsequently influence their attitude towards purchasing environmentally-friendly products like LED lights. Reminding and educating Malaysians on the influence of using LED lighting and renewable energy will influence their attitude to use such products. More efficient energy usage is achievable with changes in energy consumption behavior and mindset shift.

Finally, the results showed that a positive attitude towards the use of LED lights influences the consumer’s intention to buy LED lights. This result is consistent with the finding by Paul et al. [62] that attitude has a powerful relationship with a consumer’s interest in buying green products. Attitude is an important factor because, when a customer has a favorable attitude and is more concerned about the environment, that individual is more likely to make efforts to alleviate the adverse impact of their actions [63] and form the intention to buy more environmentally-friendly products such as LED lights.

This study investigated the moderating function of anticipated pride and guilt on the relationship between attitude and the desire to buy LED lights. The results show that anticipated pride failed to support the moderating connection between attitude and the intention to buy LED lights. In the context of LED, regardless of whether the individuals feel pride or not for using the LED products, when the individuals possess positive attitude towards the LED lights, it will directly affect their intention to buy the lights. Therefore, anticipated pride does not play a moderating role. Previous studies have not found a significant relationship between the impact of anticipated pride and guilt on personal behavioral norms [4]. However, this study found that anticipated guilt plays an important role as the moderator of attitude and the intention to purchase LED lights. It is expected that individuals who experience feelings of guilt towards performing activities that affect the environment will form intentions in line with their attitudes and act in ways that contribute to environmental preservation [49]. The study results indicate that feelings of guilt affect both attitude and the intention to buy environmentally-friendly products such as LED lights.

For environmental researchers and policy-makers, the results of the study provide useful insights into consumers’ behaviors towards the adoption of LED lights, especially those behaviors of consumers in Malaysia. The direct effect of awareness on responsibility indicates the importance of enhancing consumers’ awareness about environmentally-friendly lighting such as LED lights. In addition, personal norms and attitudes toward LED lights are found to be significant factors in consumers’ decision-making processes. Hence, policy-makers should develop eco-awareness programs to project a favorable image of LED light adoption. Policy-makers could influence individuals’ personal norms and attitudes which will eventually enhance their decision-making processes. The research framework adopted in this study provides a useful tool for understanding individuals’ complicated decision-making in the usage of environmentally-friendly lighting. The proposed framework can be further improved and tested by the integration of additional factors which are relevant to the adoption of environmentally-friendly lighting.

## Conclusion

This study highlights the important factors that affect the adoption of LED lights in Malaysia, using the PLS method. The results indicate that awareness of the effects of LED lights creates a feeling of responsibility towards the adoption of LED lights. In addition, responsibility influences personal norms towards LED lights which eventually influences attitudes towards LED lights. A positive attitude towards LED lights will subsequently affect the intention to purchase LED lights. The findings of the study show that anticipated pride may not weaken the relationship between attitude and the intention to purchase LED lights, while anticipated guilt has a moderating function for both attitude and intention to buy LED lights.

The literature shows that extending the NAM through the inclusion of intentions can significantly enhance the understanding of behavioral differences [4]. In this tradition, the present study incorporated attitude and intention as variables in the NAM, and included anticipated guilt and anticipated pride as moderators of the relationship between attitude and intention. Factors already included in the NAM, namely, awareness, responsibility and personal norms, also formed part of the extended model. The study makes a contribution to theory by extending the NAM. It addresses the dearth of published work on the use of the NAM in understanding Malaysian consumers’ intention to buy LED lights.

## Supporting information

S1 Appendix Cross loadings of the constructs. (DOCX)

S1 Data (DOCX)
