# Supplementary material for: Feelings of guilt and pride: Consumer intention to buy LED lights
Source: PLoS One. 2020 Jun 25;15(6):e0234602. doi: 10.1371/journal.pone.0234602 (PMC7316250; doi:10.1371/journal.pone.0234602)
Supplement: S1 Appendix — (DOCX) [file pone.0234602.s001.docx]

**Appendix 1**

**Cross Loadings of the Constructs**

|  | **Attitude** | **Awareness** | **Intention** | **Anticipated Guilt** | **Anticipated Pride** | **Personal Norm** | **Responsibility** | |
| --- | --- | --- | --- | --- | --- | --- | --- | --- |
| **AT1** | 0.800 | 0.306 | 0.415 | 0.236 | 0.319 | 0.438 | 0.418 |  |
| **AT2** | 0.732 | 0.291 | 0.341 | 0.146 | 0.208 | 0.386 | 0.366 |  |
| **AT3** | 0.831 | 0.280 | 0.441 | 0.174 | 0.277 | 0.422 | 0.371 |  |
| **AT4** | 0.808 | 0.340 | 0.445 | 0.172 | 0.290 | 0.461 | 0.374 |  |
| **AW2** | 0.218 | 0.689 | 0.082 | 0.150 | 0.164 | 0.248 | 0.309 |  |
| **AW3** | 0.237 | 0.718 | 0.131 | 0.136 | 0.148 | 0.293 | 0.319 |  |
| **AW4** | 0.279 | 0.753 | 0.167 | 0.171 | 0.183 | 0.332 | 0.313 |  |
| **AW5** | 0.294 | 0.730 | 0.252 | 0.202 | 0.160 | 0.403 | 0.339 |  |
| **AW6** | 0.262 | 0.702 | 0.166 | 0.124 | 0.147 | 0.314 | 0.320 |  |
| **AW7** | 0.331 | 0.688 | 0.276 | 0.162 | 0.204 | 0.388 | 0.403 |  |
| **BI1** | 0.471 | 0.258 | 0.839 | 0.239 | 0.335 | 0.451 | 0.395 |  |
| **BI2** | 0.448 | 0.230 | 0.863 | 0.222 | 0.299 | 0.456 | 0.376 |  |
| **BI3** | 0.436 | 0.220 | 0.857 | 0.211 | 0.320 | 0.457 | 0.363 |  |
| **BI4** | 0.393 | 0.174 | 0.770 | 0.267 | 0.273 | 0.426 | 0.311 |  |
| **BI5** | 0.346 | 0.159 | 0.715 | 0.328 | 0.302 | 0.400 | 0.288 |  |
| **G1** | 0.228 | 0.207 | 0.319 | 0.887 | 0.503 | 0.353 | 0.333 |  |
| **G2** | 0.216 | 0.199 | 0.306 | 0.918 | 0.493 | 0.367 | 0.311 |  |
| **G3** | 0.180 | 0.189 | 0.233 | 0.875 | 0.465 | 0.280 | 0.267 |  |
| **G4** | 0.217 | 0.215 | 0.252 | 0.878 | 0.489 | 0.321 | 0.225 |  |
| **G5** | 0.172 | 0.170 | 0.238 | 0.866 | 0.425 | 0.272 | 0.226 |  |
| **P1** | 0.296 | 0.203 | 0.344 | 0.512 | 0.863 | 0.351 | 0.291 |  |
| **P2** | 0.300 | 0.245 | 0.349 | 0.504 | 0.887 | 0.354 | 0.321 |  |
| **P3** | 0.275 | 0.176 | 0.332 | 0.488 | 0.887 | 0.347 | 0.306 |  |
| **P4** | 0.316 | 0.176 | 0.288 | 0.378 | 0.813 | 0.303 | 0.277 |  |
| **P5** | 0.303 | 0.211 | 0.295 | 0.404 | 0.823 | 0.305 | 0.313 |  |
| **PN1** | 0.334 | 0.309 | 0.340 | 0.275 | 0.241 | 0.686 | 0.448 |  |
| **PN2** | 0.496 | 0.372 | 0.469 | 0.254 | 0.343 | 0.786 | 0.472 |  |
| **PN3** | 0.327 | 0.386 | 0.378 | 0.289 | 0.246 | 0.771 | 0.420 |  |
| **PN4** | 0.437 | 0.330 | 0.420 | 0.276 | 0.326 | 0.743 | 0.410 |  |
| **R1** | 0.415 | 0.444 | 0.354 | 0.238 | 0.327 | 0.434 | 0.768 |  |
| **R2** | 0.299 | 0.347 | 0.264 | 0.151 | 0.196 | 0.365 | 0.718 |  |
| **R3** | 0.240 | 0.205 | 0.234 | 0.242 | 0.181 | 0.339 | 0.638 |  |
| **R4** | 0.407 | 0.342 | 0.369 | 0.276 | 0.289 | 0.535 | 0.776 |  |
