# Supplementary material for: Feelings of guilt and pride: Consumer intention to buy LED lights
Source: PLoS One. 2020 Jun 25;15(6):e0234602. doi: 10.1371/journal.pone.0234602 (PMC7316250; doi:10.1371/journal.pone.0234602)
Supplement: S1 Data — (DOCX) [file pone.0234602.s002.docx]

***
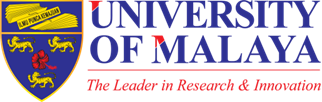
***

***Socio Economic Impacts of Light-Emitting Diode (LED) Lighting Usage***

*
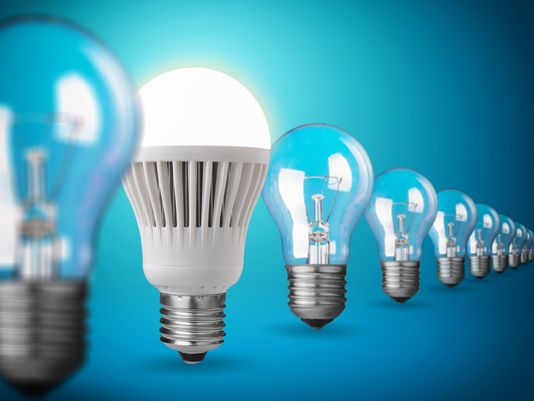

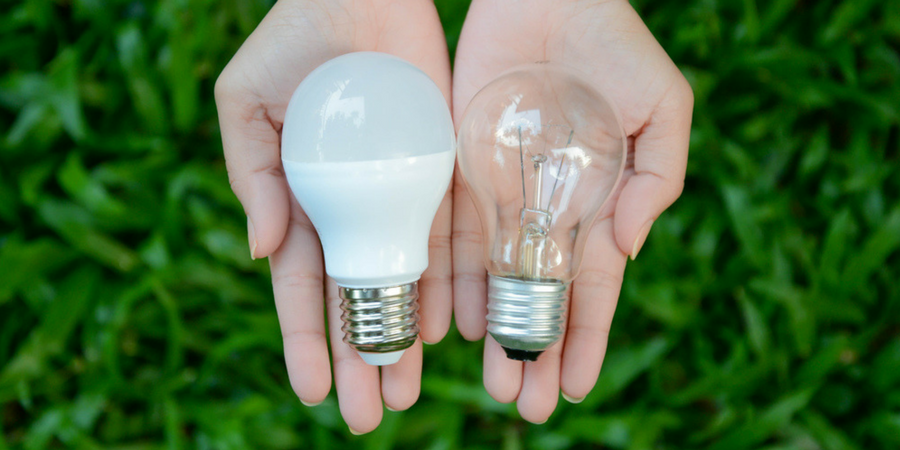
*

LED lights are environmental friendly lights as they consume less energy compare to conventional (normal) lights and they are not including lead or mercury. While they are easier to install they have longer life and durability. These lights produce less heat and cause the electricity bill to decrease.

*Dear Respondents,*

*The objective of this research is to study the LED light usage among Malaysian households. The research will examine the awareness about LED lights among Malaysians. The study will also investigate the antecedents influencing LED usage. The expected outcome will provide a full study and reports for LED usage and growth in Malaysia from residential users’ perspective. The study findings will allow industry players to plan their strategies to increase LED consumption in the country. This survey would take approximately less than 15 minutes to be completed. Please answer all the questions as honestly as possible and return the completed questionnaires to us.*

***All the information provided will be treated as confidential and will not be disclosed to any commercial or external parties. The data will be analyzed and used in the aggregate form only.***

*Thank you for your kind cooperation and valuable assistance participating in this survey.*

*Queries pertaining to this questionnaire can be directed as stated below:*

**Section A:** Please indicate your level of agreement for the following statements on the scale (1 to 5) where 5 represents “strongly agree” and 1 represents “strongly disagree” (kindly circle only one number for each item).

| **NO.** | **QUESTIONS** | **Strongly Disagree** | **Disagree** | **Neutral** | **Agree** | **Strongly Agree** |
| --- | --- | --- | --- | --- | --- | --- |
| Intention  BI1 | I intend to use LED in the next 6 months. | 1 | 2 | 3 | 4 | 5 |
| BI2 | I predict that I would use LED in the next 6 months. | 1 | 2 | 3 | 4 | 5 |
| BI3 | I plan to use LED in next 6 months. | 1 | 2 | 3 | 4 | 5 |
| BI4 | I would buy LED lights/lamps even if they are more expensive than other products. | 1 | 2 | 3 | 4 | 5 |
| BI5 | I am willing to buy LED lights/lamps even if they are less readily available in shops. | 1 | 2 | 3 | 4 | 5 |
| BI6 | If I find that LED lights/lamps are more expensive and difficult to get, it would stop me from buying and using them. | 1 | 2 | 3 | 4 | 5 |
| Awareness  AW1 | Conventional lights (normal lights) can cause air pollution and global warming derived from energy use. | 1 | 2 | 3 | 4 | 5 |

| AW2 | The effects of pollution from conventional lights (normal lights) on public health are worse than we realize. | 1 | 2 | 3 | 4 | 5 |
| --- | --- | --- | --- | --- | --- | --- |
| AW3 | Pollution generated in one country harms people all over the world. | 1 | 2 | 3 | 4 | 5 |
| AW4 | Conventional lights (normal lights) cause environmental deteriorations (e.g., excessive use of energy resources). | 1 | 2 | 3 | 4 | 5 |
| AW5 | The balance in nature is delicate and easily upset. | 1 | 2 | 3 | 4 | 5 |
| AW6 | Over the next several decades, thousands of species will become extinct. | 1 | 2 | 3 | 4 | 5 |
| AW7 | An environmentally responsible light practicing energy conservation and diverse green activities helps to minimize environmental degradation. | 1 | 2 | 3 | 4 | 5 |
| Responsibility  R1 | By using LED light, I contribute to better air quality. | 1 | 2 | 3 | 4 | 5 |
| R2 | In principle, one person alone cannot contribute to better air quality. | 1 | 2 | 3 | 4 | 5 |
| R3 | Whether or not I pollute the air is beyond the realm of personal choice. | 1 | 2 | 3 | 4 | 5 |
| R4 | I intend to reduce air pollution by using LED lights. | 1 | 2 | 3 | 4 | 5 |
| **Personal Norm**  PN1 | Due to my personal values I feel obliged to use conventional lights (normal lights) as seldom as possible. | 1 | 2 | 3 | 4 | 5 |
| PN2 | No matter what other people do, my own values tell me that it is right to use the LED lights. | 1 | 2 | 3 | 4 | 5 |
| PN3 | Using the environmentally damaging light would be against my personal values. | 1 | 2 | 3 | 4 | 5 |
| PN4 | Due to my own values I feel personally obliged, to use environmentally friendly lights like LEDs. | 1 | 2 | 3 | 4 | 5 |
| **Attitude**  AT1 | Using LED lights is a good idea. | 1 | 2 | 3 | 4 | 5 |
| AT2 | Using LED lights is a wise idea. | 1 | 2 | 3 | 4 | 5 |
| AT3 | I would like to use LED lights. | 1 | 2 | 3 | 4 | 5 |
| AT4 | Using LED lights is pleasant. | 1 | 2 | 3 | 4 | 5 |

**Section B:** Please indicate your level of feelings for the following statements on the scale (1 to 5) where 5 represents “Extremely” and 1 represents “Not at All” (kindly circle only one number for each item).

- Imagine that you are in a store and decide not to buy LED lights. How would you feel?

| **NO.** | **QUESTIONS (Guilt)** | **Not at All** | **Slightly** | **Somewhat** | **Very Much** | **Extremely** |
| --- | --- | --- | --- | --- | --- | --- |
| G1 | Guilty | 1 | 2 | 3 | 4 | 5 |
| G2 | Remorseful | 1 | 2 | 3 | 4 | 5 |
| G3 | Sorry | 1 | 2 | 3 | 4 | 5 |
| G4 | Bad | 1 | 2 | 3 | 4 | 5 |
| G5 | Ashamed | 1 | 2 | 3 | 4 | 5 |

- Imagine that you are in a store and decide to buy LED lights. How would you feel?

| **NO.** | **QUESTIONS (Pride)** | **Not at All** | **Slightly** | **Somewhat** | **Very Much** | **Extremely** |
| --- | --- | --- | --- | --- | --- | --- |
| P1 | Proud | 1 | 2 | 3 | 4 | 5 |
| P2 | Accomplished | 1 | 2 | 3 | 4 | 5 |
| P3 | Confident | 1 | 2 | 3 | 4 | 5 |
| P4 | Satisfied | 1 | 2 | 3 | 4 | 5 |
| P5 | Worthwhile | 1 | 2 | 3 | 4 | 5 |

**Section D:** Please answer the following questions by either filling in the spaces provided or ticking the boxes.

1. Please indicate your gender:

□ Male □ Female

1. Which age group do you belong to?

□ Below 21 □ 21-30 □ 31-40 □ 41-50 □ Above 50

1. Please indicate your highest academic qualification?

□ Primary Level (UPSR)

□ Secondary Level (PMR, SPM,STPM)

□ Diploma

□ Bachelor’s Degree

□ Master’s Degree/Ph.D

4. Please indicate the type of house you are currently living:

□ Bungalow/Villas

□ Apartment /Flat

□ Condominium/Residence, Suite

□ Duplex/Double Storey

□ Shop Houses

Thank you for taking the time to complete this survey!
